# Prevention of Post-Operative Pain after Elective Brain Surgery: A Meta-Analysis of Randomized Controlled Trials

**DOI:** 10.3390/medicina59050831

**Published:** 2023-04-24

**Authors:** Giorgio Fiore, Edoardo Porto, Mauro Pluderi, Antonella Maria Ampollini, Stefano Borsa, Federico Giuseppe Legnani, Davide Giampiccolo, Anna Miserocchi, Giulio Andrea Bertani, Francesco DiMeco, Marco Locatelli

**Affiliations:** 1 Unit of Neurosurgery, Foundation IRCCS Ca’ Granda Ospedale Maggiore Policlinico, 20122 Milan, Italy; 2 Department of Pathophysiology and Transplantation, University of Milan, 20122 Milan, Italy; 3 Department of Neurosurgery, National Hospital for Neurology and Neurosurgery, London WC1N 3BG, UK; 4 Department of Neurosurgery, Fondazione IRCCS Istituto Neurologico C. Besta, 20133 Milan, Italy; 5 Department of Neurosurgery, School of Medicine, Emory University, Atlanta, GA 30322, USA; 6 Institute of Neuroscience, Cleveland Clinic London, Grosvenor Place, London SW1X 7HY, UK; 7 Department of Clinical and Experimental Epilepsy, UCL Queen Square Institute of Neurology, University College, London WC1E 6BT, UK; 8 Department of Neurosurgery, Johns Hopkins University, Baltimore, MD 21205, USA

**Keywords:** pain, craniotomy, acute pain, brain surgery, post-operative pain, treatment, prevention, management, brain surgery, headache

## Abstract

*Background and Objective:* To analyze the effects of several drug for pain prevention in adults undergoing craniotomy for elective brain surgery. *Material and Methods:* A systematic review and meta-analysis were conducted in accordance with the Preferred Reporting Items for Systematic Reviews and Meta-Analyses (PRISMA) 2020 guidelines. The inclusion criteria were limited to randomized controlled trials (RCTs) that evaluated the effectiveness of pharmacological treatments for preventing post-operative pain in adults (aged 18 years or older) undergoing craniotomies. The main outcome measures were represented by the mean differences in validated pain intensity scales administered at 6 h, 12 h, 24 h and 48 h post-operatively. The pooled estimates were calculated using random forest models. The risk of bias was evaluated using the RoB2 revised tool, and the certainty of evidence was assessed according to the GRADE guidelines. *Results:* In total, 3359 records were identified through databases and registers’ searching. After study selection, 29 studies and 2376 patients were included in the meta-analysis. The overall risk of bias was low in 78.5% of the studies included. The pooled estimates of the following drug classes were provided: NSAIDs, acetaminophen, local anesthetics and steroids for scalp infiltration and scalp block, gabapentinoids and agonists of adrenal receptors. *Conclusions:* High-certainty evidence suggests that NSAIDs and acetaminophen may have a moderate effect on reducing post-craniotomy pain 24 h after surgery compared to control and that ropivacaine scalp block may have a bigger impact on reducing post-craniotomy pain 6 h after surgery compared to control. Moderate-certainty evidence indicates that NSAIDs may have a more remarkable effect on reducing post-craniotomy pain 12 h after surgery compared to control. No moderate-to-high-certainty evidence indicates effective treatments for post-craniotomy pain prevention 48 h after surgery.

## 1. Introduction

Post-operative pain is a crucial clinical issue that has generated increased attention in recent years [1]. Despite the aim of contemporary anesthesiology to keep patients free from experiencing pain, it was not until 1996, when De Benedittis et al. conducted a pilot study to assess post-operative pain in neurosurgery, that the extent, intensity, and duration of acute pain experienced by neurosurgical patients undergoing brain surgery were quantified [2,3].

Craniotomy is the most frequent cause of headache and pain for almost all types of elective cranial neurosurgical pathologies [4,5]. Acute post-operative pain often causes alarm, concern, anxiety, doubt in treatment, a sense of failure and even depression in neurosurgical patients, frequently leading to chronic pain and headache [6]. In this context, craniotomy-related headache has gained so much importance that it has been recognized as a specific nosological class by the Committee of the International Headache Society, being defined as post-craniotomy headache with outset within 7 days after craniotomy and lasting for less than 3 months [7]. Notably, the incidence of post-operative pain largely hinges on the chosen surgical approach [8].

Post-craniotomy pain is mainly somatic and originates from the scalp, peri-cranial muscles and soft tissues [9,10,11]. The manipulation of dura mater during surgery also activates its pathways [2]. The mechanical stimulation caused by the incision and traction of cranial tissues triggers nervous terminals and specific nociceptors, resulting in post-operative pain [12]. As a consequence, it is commonly localized to the incision site and the surrounding area, while diffuse headache can stem from the dura mater [13,14,15].

The nature of pain after craniotomy is usually pulsating or pounding [16]. Constant and continuous pain is less frequently seen. The long-term persistence of post-surgical pain after craniotomy is observed in many patients, with proposed pathogenetic hypotheses, including dura mater irritation, peri-cranial muscle stiffness and retraction, cerebrospinal fluid hypotension and aseptic meningitis [17]. Patient positioning during surgery may also result in long-term tension headaches and neck muscle spasms of a possible muscular origin [16]. Interestingly, more than 45% of post-craniotomy patients may experience headache during dental evaluation [14]. These patients showed tenderness around the masseter muscles, with opening the mouth and jaw protrusion triggering the headache [14].

The consequences of pain after brain surgery can be severe and long-standing, including risks of post-operative hemorrhage due to arterial hypertension, prolonged recovery time, and an increased risk of long-term headaches [18,19]. Several studies have analyzed the effect of various drugs in reducing the incidence of pain for these patients [20]. While there is now more evidence regarding pain reduction options for adults undergoing elective brain surgery, it is still uncertain which options are more effective [21,22,23,24,25]. Currently, there is evidence that NSAIDs can post-operatively reduce pain for up to 24 h, while the evidence for pain reduction with other drug classes, such as acetaminophen, dexmedetomidine, pregabalin or gabapentin, local anesthetics for scalp block and scalp infiltration, is less certain and of low-to-moderate quality.

The aim of this meta-analysis is to analyze the effect of several drug classes to provide the best chance of reducing pain for adults undergoing craniotomy for elective brain surgery by collecting and synthesizing the results of randomized clinical trials that investigated pain-relieving drugs for this patient group, including both invasive and non-invasive routes of administration. To accurately address this research question, only studies conducted following an approved high standard were included, and a risk of bias assessment was conducted to ensure the validity of the results. Studies from various countries was analyzed to gather comprehensive and relevant information.

## 2. Methods

This systematic review with meta-analysis followed the Preferred Reporting Items for Systematic Reviews and Meta-Analyses (PRISMA) 2020 guidelines [26,27].

### 2.1. Type of Studies and Population

The inclusion criteria for this systematic review and meta-analysis were limited to randomized controlled trials (RCTs) that evaluated the effectiveness of pharmacological treatments for preventing post-operative pain in adults (aged 18 years or older) undergoing craniotomies. Non-randomized trials, observational retrospective and prospective studies, case reports and review articles were excluded to reduce the inaccuracies associated with different study designs, non-random allocation, the absence of control groups and varying inclusion and exclusion criteria. The decision to exclude the pediatric population was based on concerns regarding the accuracy of pain assessment in pediatric patients compared to that of adults, and the higher likelihood of different pain thresholds between adult and pediatric populations.

### 2.2. Type of Intervention

This meta-analysis considered any kind of pharmacological treatment for the prevention of post-operative pain in adult patients undergoing craniotomies. The aim was to evaluate the efficacy of these treatments in reducing the incidence and intensity of post-operative pain. Non-preventive pharmacological treatments, such as patient-controlled analgesia (PCA), or non-pharmacological treatments, such as acupuncture and protocols for patient mobilization, were excluded from this meta-analysis.

### 2.3. Outcome Measure

The main outcome measures were represented by mean differences in validated pain intensity scales, such as the Visual Analogue Scale (VAS) or the Numerical Rating Scale (NRS), being post-operatively administered at 6 h, 12 h, 24 h and 48 h. If a time point was missing, the closest available one was considered as long as it did not differ by more than 2 h. For studies reporting VAS intervals between 0 and 100, the pain intensity VAS was rescaled to an interval from 0 to 10. Studies were not included in this meta-analysis if the assessment of post-operative pain was indirectly addressed through the registration and report of analgesic drugs’ consumption, such as post-operative consumption of opioids, or through the analysis of hemodynamic parameters usually related to pain, such as arterial blood pressure or heart rate.

### 2.4. Information Sources

The following databases and registers were independently screened by two reviewers (G.F. and E.P.): PubMed, CENTRAL and ClinicalTrials.gov. The initial search was concluded on 20 November 2022. We conducted an updated search on 30 December 2022. A third search stage consisted of examining any other pertinent trial being included in the reference lists of relevant articles. This third phase was jointly conducted by two reviewers.

### 2.5. Search Strategy

The following research terms were used to identify relevant articles: craniotomy, pain, post-operative pain, prevention, treatment and management, along with their MeSH terms. Only articles written in English were included in this investigation, and no filters were applied during the research process.

### 2.6. Selection Process

Two authors (G.F. and E.P.) independently reviewed the titles and abstracts of all retrieved articles, categorizing them as included, excluded or maybe. Shortly, studies that did not meet the inclusion criteria were excluded at this stage (e.g., studies on animals, studies not in English, case reports, in vivo studies, etc.). In the case of disagreement, the authors reached consensus through full-text review and discussion. If necessary, a third author (G.A.B.) was consulted. Next, two authors reviewed the full texts of all the initially included articles to assess their eligibility. Again, in case of disagreement, the consensus was reached by discussion or by consulting the third author. No automatic tools were employed during the articles’ selection process.

### 2.7. Data Collection

Two reviewers (G.F. and E.P.) extracted data from the included articles. As per continuous outcome measures, we calculated the mean and standard deviations (SD). Where a scale from 0 to 100 was employed, we rescaled it to a scale from 0 to 10. If the dispersion of the outcome measure was reported as median, first and third quartiles, interquartile range (IQR) and/or minimum and maximum values, the mean and SD were calculated using the formula proposed by the Tong group [28,29] and also suggested in Chapter 6 of the *Cochrane Handbook for Systematic Reviews of Interventions* (Higgins 2022) [30]. If SDs were not provided, we estimated them from the confidence intervals (CIs) and standard errors (SEs) where available [30]. If only the *p* value was usable, the SD was estimated through the t statistic, and then through the estimated SD, as described in the *Cochrane Handbook for Systematic Reviews of Interventions* (Higgins 2022). If none of the aforementioned values was reported, the SD was estimated as the mean of the SDs of the included studies in this meta-analysis at that time point. If a study reported more than one intervention arm and both of them were deemed to be suitable for inclusion in the meta-analysis, the control group was divided between the two arms.

### 2.8. Heterogeneity

The clinical heterogeneity was assessed by evaluating the differences in the patient population, type of intervention, administration method, dosages and outcome measures. The methodological heterogeneity was assessed by evaluating the risk of bias (RoB). The statistical heterogeneity was evaluated through Cochran’s Q (χ^2^) test, *I*^2^, tau^2^ and prediction intervals. In case of moderate-to-high heterogeneity, the pooled estimates were checked for outlier cases and influence analyses were conducted. We planned to perform subgroup analyses if the synthesis included the same class of drugs, but different agents and/or infratentorial and supratentorial craniotomies.

### 2.9. Risk of Bias Assessment

Two authors (E.P. and G.F.) independently assessed the risk of bias in included studies using the RoB2 revised tool for assessing the risk of bias in randomized trials [31]. Any discrepancies were analyzed and solved by discussion. For each included primary study, the following seven domains were assessed:

1. Randomization process;
2. Effect of assignment to intervention;
3. Effect of adhering to intervention;
4. Missing outcome data;
5. Measurement of the outcome;
6. Selection of the reported result;
7. Overall risk of bias.

For each domain, the risk of bias was determined as low, there being some concerns or high according to methods used to ensure the minimization of each form of bias:

1. Low: where information was available that clearly demonstrated that efforts were made to ensure that there was minimal bias in that domain and the described methods were robust enough to have a high likelihood of being effective.
2. Some concerns: when the information available was insufficient to be confident that the method used to minimize bias was robust enough to be effective.
3. High: when the study did not report any method to minimize bias in that domain.
4. In the present study, unblinded, single-blinded and double-blinded studies were included to provide a comprehensive summary of the overall available evidence. Thus, a greater level of overall ‘performance’ bias was tolerated than that which would have occurred if double-blinded studies were the only ones to be included.

### 2.10. Reporting Bias Assessment

Publication biases were examined by funnel plots inspections if more than 5 articles were available (see GRADE guidelines), as well as by direct comparison of estimates obtained through both the random effects and fixed effect models. The latter approach, which is suggested in the *Cochrane Handbook for Systematic Reviews of Interventions*, allows researchers to hypothesize a reporting bias related to missing small studies. By screening the main databases and registers, the authors tried to reduce the risks of suitable studies being missed, consequently reducing the risks of reporting biases. Finally, the lag bias (suggested by the early publication of positive results) was also explored.

### 2.11. Synthesis Methods

We planned to calculate the pooled estimates if at least two studies would have been available for the synthesis. If the included studies utilized the same scale for pain assessment, a mean difference (MD) effect size (Hedges’ *g*) and its confidence intervals (CIs) for continuous pairwise data were used. In the case of different pain scales being employed, a standardized mean difference (SMD) and its CIs were applied. We did not calculate any pooled estimates if the included studies showed significant clinical or methodological heterogeneity. We performed meta-analysis using R software 4.2.2 (R Foundation for Statistical Computing, Vienna, Austria; http://www.r-project.org/index.html (accessed on 1 September 2022)). A random forest model was employed to address both sampling error and other sources of variance (such as between-study heterogeneity). We used a restricted maximum likelihood (RML) estimator [32] to assess between-study heterogeneity (*t*^2^). This choice related to the kind of outcome measure, which was continuous, and to the expected important variance in the sample size among the included studies (see Veroniki et al. [33]). We used Knapp–Hartung (KH) adjustments to calculate the confidence interval around the pooled effect [34]; in the case of very homogeneous effects, KH adjustments were not employed. We categorized the main intervention groups based on the drug class being investigated (such as steroids, no-steroidal anti-inflammatory drugs (NSAIDs), acetaminophen, gabapentinoids, local anesthetics for scalp infiltration, local anesthetics for scalp block, etc.). We used forest plots to visually display the results of the syntheses. For each study, the observed effect, CIs and the weight of the study were reported. Moreover, the overall pooled effect and measures of between-study heterogeneity were described.

### 2.12. Sensitive Analysis

We planned to conduct sensitive analyses based on the risk of bias in the studies included in the meta-analysis. This approach was based on providing a comprehensive understanding of the evidence, including the overall evidence and evidence based on the risk of bias, so that decisions could be made based on accurate and trustworthy information.

### 2.13. Certainty Assessment

Two authors (G.F. and E.P.) jointly assessed the certainty of evidence according to GRADE guidelines [35]. As described in Chapter 5 of the *GRADE Handbook*, the following domains were used for downgrading the quality of evidence: (1) limitations in study design and execution (risk of bias), (2) inconsistency of results, (3) indirectness of evidence, (4) imprecision and (5) publication bias. The quality of the evidence was upgraded based on the following factors: (1) the large magnitude of the effect, (2) the dose–response gradient and (3) the plausible confounding effect. We graded the certainty of evidence as high, moderate, low and very low according to GRADE guidelines [36,37,38]. Summary of findings’ tables were built using GRADEpro GDT software (https://www.gradepro.org (accessed on 1 January 2023)).

## 3. Results

**Figure 1:**
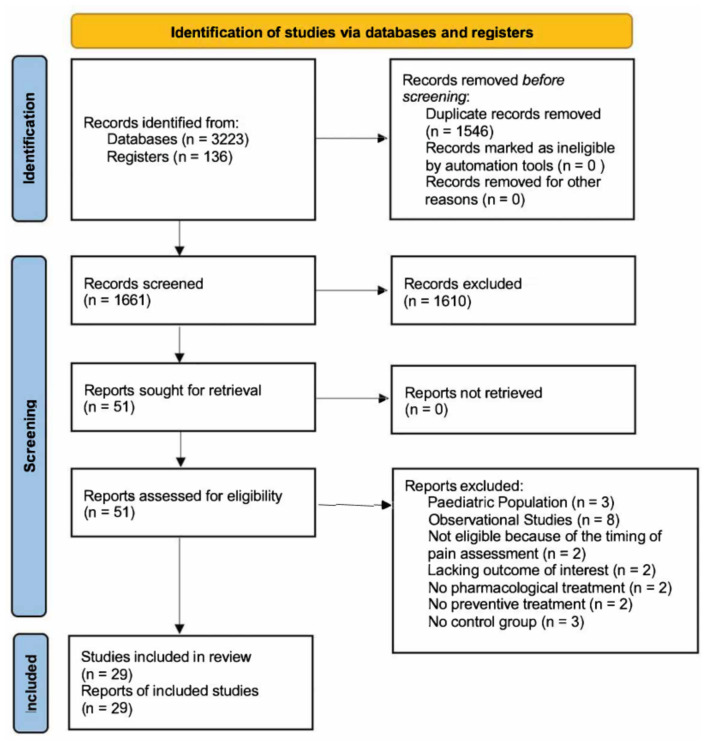
Flow diagram according to the PRISMA guidelines.

The flow diagram according to the PRISMA guidelines is available in Figure 1. We found 3223 records through database searching and 136 records via register searching. After the removal of duplicates, 1661 records were screened by assessing titles and abstracts. Fifty-one full texts were assessed for eligibility in this meta-analysis. Three studies were excluded since they analyzed the effects of preventive treatment for post-craniotomy pain in the pediatric population [39,40,41]. Eight studies were excluded because they were determined to be observational studies [42,43,44,45,46,47,48,49,50]. Two studies were excluded because their post-operative assessment was limited to only the first post-operative hour and did not match any of the four time intervals we planned to analyze [51,52]. Two studies were excluded since they did not include the outcome of interest for inclusion in this meta-analysis. Particularly, Jose et al. evaluated the time for the first dose of analgesic post-operatively required, and Theerth et al. evaluated the analgesia nociception index during skull pin application [53,54]. Two studies were excluded because they reported the effects of non-pharmacological treatments (acupuncture) on post-craniotomy pain [55,56]. Two studies were excluded since they did not analyze the impact of preventive treatments on post-craniotomy pain [57,58]. Three studies did not include a control group and were excluded from this meta-analysis [59,60,61]. In total, 29 studies and 2376 patients were included in the systematic review [62,63,64,65,66,67,68,69,70,71,72,73,74,75,76,77,78,79,80,81,82,83,84,85,86,87,88,89,90].

### 3.1. Patient Population

The patients in this meta-analysis underwent elective cranial surgeries and had an ASA score mainly between I and III. Nine studies (31%) did not specify the ASA score of the patients. The pain was measured using the following scales: 10 studies used the Numeric Rating Scale (NRS), 18 used the Visual Analogue Scale (VAS) and 1 study used the Visual Numeric Scale (VNS). All patients received a general anesthetic technique. Nineteen studies embedded a purely supra-tentorial craniotomy patient population, while six studies reported the pain outcomes of a mixed cohort of patients undergoing supra-tentorial or infra-tentorial craniotomies. Four studies did not classify the patient population according to the craniotomy region. Table S1 (Supplementary Material) provides a summary of the patient population features.

### 3.2. Risk of Bias

**Figure 2:**
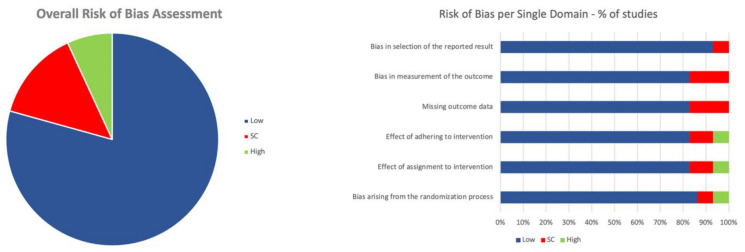
Risk of bias.

For each included study, a detailed ‘Risk of bias’ assessment is provided in Table S2 (Supplementary Materials). A graphical representation of both the overall and the single-domain risk of bias for the included studies is provided in Figure 2. A low overall risk of bias was identified in 78.5% of the included studies (22/28), with four studies having some concerns (14.2%) and two studies having a high risk of bias (7%).

### 3.3. Interventions

#### 3.3.1. NSAIDs

**Figure 3:**
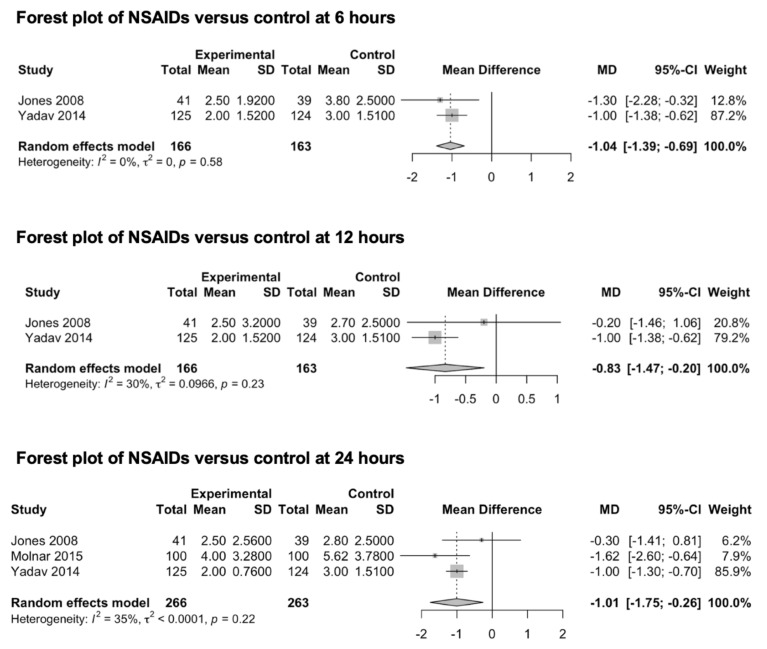
Forest plot of NSAIDs versus control at 6, 12 and 24 h [62,63,64]. As per convention, the squares in the forest plots represent the study weight, while the diamonds represent the pooled effect.

Three studies (529 patients) that were included in this meta-analysis explored the preventive effect of NSAIDs on post-craniotomy pain (Figure 3) [62,63,64]. Two studies [63,64] reported the effects of diclofenac (from 50 to 100 mg) and one study [62] reported those of parecoxib (40 mg). In two studies, NSAIDs were orally administered [63,64], while in Jones et al.’s study, parecoxib was intravenously administered. Two studies [62,64] reported the pain outcomes at 6 and 12 h after surgery, while three studies [62,63,64] reported the main outcomes 24 h after surgery. Only one study reported pain outcomes at 48 h [64]. The pooled estimates (MD) were −1.04 (CIs: −1.39; −0.69; *I*^2^ = 0%; *p* < 0.0001) at 6 h, −0.83 (CIs: −1.47; −0.20; *I*^2^ = 30%; *p* = 0.01) at 12 h and −1.01 (CIs: −1.75; −0.26; *I*^2^ = 35%; *p* = 0.03) at 24 h.

- Subgroup Analysis

No subgroup analyses were performed as none of the included studies explored the impact of craniotomy location (supra- versus infra-tentorial). Additionally, since only one study examined the analgesic effect of parecoxib, a subgroup analysis of the different agents could not be conducted.

#### 3.3.2. Acetaminophen

**Figure 4:**
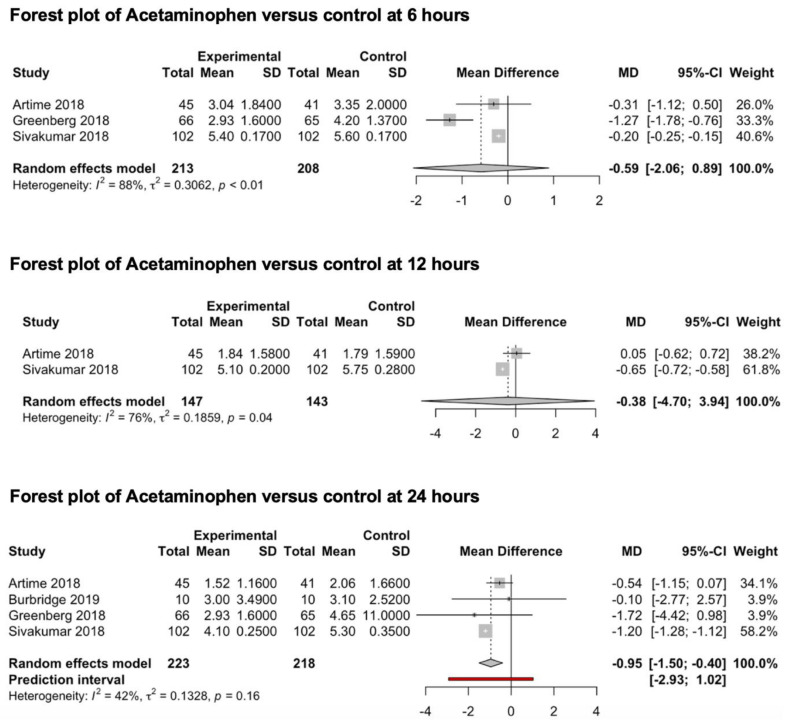
Forest plot of acetaminophen versus control at 6, 12 and 24 h [65,66,67,68].

Four studies (451 patients) included in this meta-analysis explored the preventive analgesic value of acetaminophen on post-craniotomy pain (Figure 4). Three studies [65,66,67] reported pain outcomes 6 h after surgery, two studies [65,67] reported pain outcomes 12 h after surgery and four studies [65,66,67,68] reported pain outcomes 24 h after surgery. Only one study [67] reported pain outcomes 48 h after surgery. In all four studies, 1000 mgs of acetaminophen were administered intravenously, while the control group received a saline solution. The pooled estimates (MD) were −0.59 (CIs: −2.06; 0.89; *I*^2^ = 88%; *p* = 0.23) at 6 h, −0.38 (CIs: −4.7; 3.94; *I*^2^ = 76%) at 12 h and −1.01 (CIs: −1.75; −0.26; *I*^2^ = 42%) at 24 h.

- Subgroup Analysis

No subgroup analyses were conducted since none of the included studies investigated the impact of craniotomy location (supra- versus infra-tentorial).

#### 3.3.3. Scalp Block

**Figure 5:**
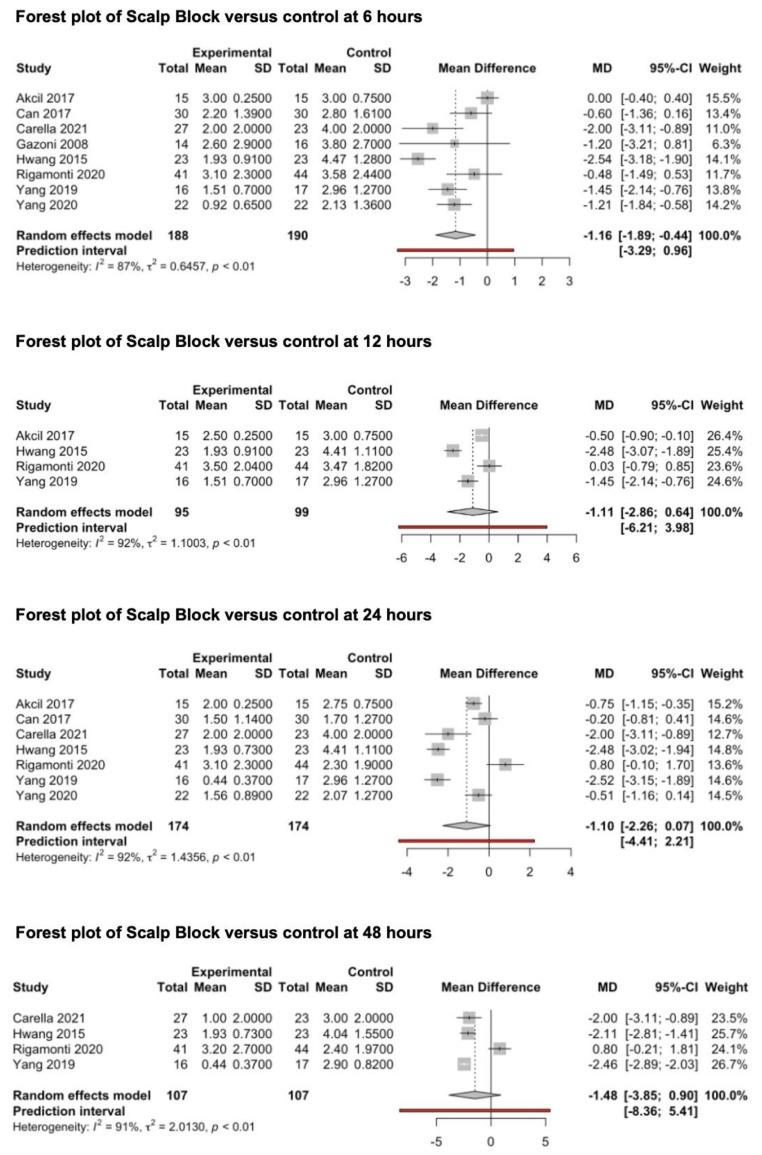
Forest plots of scalp block versus control at 6, 12, 24 and 48 h [69,70,71,72,73,74,75,76].

Eight studies (388 patients) that evaluated the preventive analgesic effect of scalp block on post-craniotomy pain were included in this meta-analysis (Figure 5) [69,70,71,72,73,74,75,76]. All studies reported the main outcomes 6 h after surgery, four studies [69,73,74,75] reported pain outcomes 12 h after surgery, seven studies [69,70,71,73,74,75,76] reported pain outcomes 24 h after surgery and four studies [71,73,74,75] reported pain outcomes 48 h after surgery. Scalp blocks were performed using bupivacaine (0.5%) in three studies [69,70,74], levobupivacaine (0.33–0.75%) in two studies [71,73] and ropivacaine (0.2–0.75%) in three studies [72,75,76]. The pooled estimates (MD) were −1.16 (CIs: −1.90; −0.44; *I*^2^ = 87%; *p* = 0.007) at 6 h, −1.11 (CIs: −2.86; 0.64; *I*^2^ = 92%; *p* = 0.14) at 12 h, −1.1 (CIs: −2.26; 0.07; *I*^2^ = 92%; *p* = 0.06) at 24 h and −1.48 (CIs: −3.86; 0.9; *I*^2^ = 91%; *p* = 0.14) at 48 h.

- Subgroup Analysis

We performed a subgroup analysis of the type of drug being used among the different RCTs (bupivacaine, levobupivacaine and ropivacaine). The pooled MDs were available at 6 and 24 h after surgery for all three classes of drugs. The pooled MD of subgroup analysis results at 6 h (p_subgroup_ < 0.0001) were −1.3 (CIs: −1.67; −0.95; *I*^2^ = 0%) for ropivacaine, −2.4 (CIs: −5.4; 0.56; *I*^2^ = 0%) for levobupivacaine and −0.2 (CIs: −1.0712; 0.6; *I*^2^ = 12%) for bupivacaine. The pooled MD of subgroup analysis results at 24 h (p_subgroup_ < 0.0001) were −1.5 (CIs: −14; 11; *I*^2^ = 95%) for ropivacaine, −2.4 (CIs: −4.8; 0.2; *I*^2^ = 0%) for levobupivacaine and −0.1 (CIs: −2; 1.8; *I*^2^ = 80%) for bupivacaine. Summarizing forest plots are available in the Supplementary Materials (Figure S1). A pooled analysis of the analgesic value of preoperative vs postoperative scalp block could not be performed since only one study investigated this aspect [77].

No subgroup analysis of the craniotomy location was conducted since none of the included studies investigated the impact of supra- versus infra-tentorial craniotomy.

b Reporting Bias

The examination of the funnel plots in Supplementary Materials (Figure S2a) suggested the presence of mild asymmetry due to the reported MD and SD in the study conducted by Hwang et al. [73], while the direct comparison of the pooled estimates at 6 h, which were obtained through both the random effects (−1.16; CIs: −1.90; −0.44; *I*^2^ = 87%; *p* = 0.007) and fixed effect models (−0.91; CIs: −1.16; −0.67; *I*^2^ = 87%; *p* < 0.0001), suggested a concordant positive effect of scalp block for pain prevention at 6 h after surgery. According to the latter finding, the presence of reporting bias related to missing small studies for the pain outcomes at 6 h was not highly suspected (see *Cochrane Handbook for Systematic Reviews of Interventions*).

The examination of the funnel plots in the Supplementary Materials (Figure S2B) suggested the presence of important asymmetry. Likewise, the discordance between the pooled estimates at 24 h obtained through the random effects (−1.1; CIs: −2.26; 0.07; *I*^2^ = 92%; *p* = 0.06) and fixed effect models (−1.13; CIs: −1.3; −0.09; *I*^2^ = 92%; *p* < 0.0001) was highly suggestive of the presence of reporting bias.

#### 3.3.4. Scalp Infiltration

**Figure 6:**
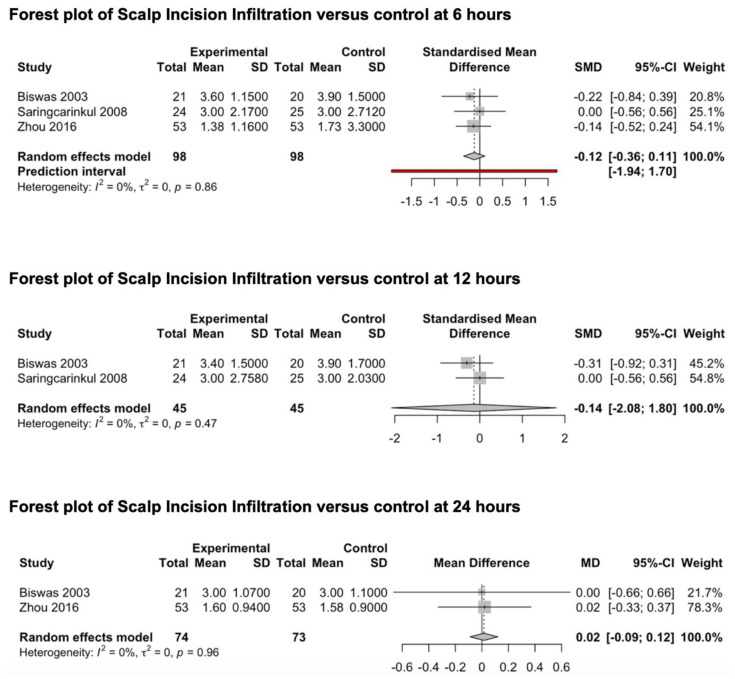
Forest plot of scalp incision infiltration versus control at 6, 12 and 24 h [78,79,80].

Three studies [78,79,80] reporting the preventive analgesic effect of scalp infiltration on post-craniotomy pain were included in this meta-analysis (Figure 6). All studies explored the effects of scalp infiltration at 6 h after surgery, two studies [78,79] explored the effects of scalp infiltration at 12 and 24 h, while only one study [78] explored the outcome of interest at 48 h. Scalp infiltration was performed through the injection of ropivacaine (0.5%) in one study [80] and bupivacaine (0.25–0.5%) together with adrenaline in the other two studies [78,79]. The pooled estimates (SMD) were −0.12 (CIs: −0.36; 0.11; *I*^2^ = 0%; *p* = 0.15) at 6 h and −0.14 (CIs: −2.1; 1.8; *I*^2^ = 0%; *p* = 0.5) at 12 h. The pooled estimate (MD) was 0.016 (CIs: −0.09; 0.12; *I*^2^ = 0%; *p* = 0.3) at 24 h.

- Subgroup Analysis

No subgroup analyses were conducted since all the included studies investigated the role of scalp infiltration in a patient population of supra-tentorial craniotomies. Since only one study investigated the analgesic effect of scalp infiltration with ropivacaine, we were unable to conduct a subgroup analysis of the different agents. A pooled analysis of the analgesic value of preoperative vs postoperative scalp infiltration could not be performed since only one RCT investigated this aspect [81].

#### 3.3.5. Gabapentinoids

**Figure 7:**
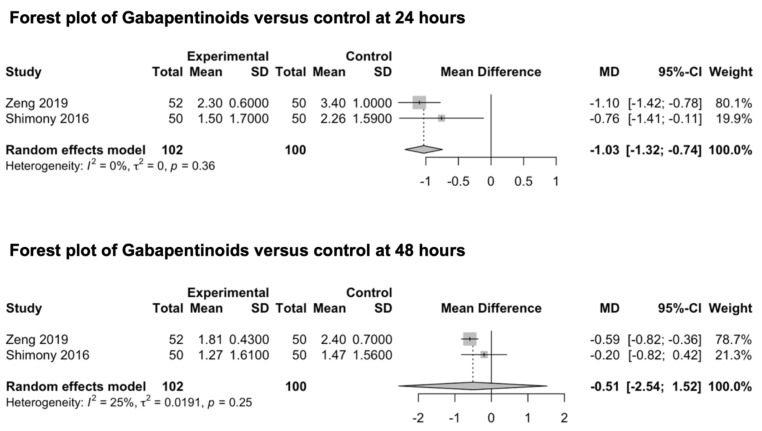
Forest plot of gabapentinoids versus control at 24 and 48 h [82,83].

Two studies investigating the preventive analgesic effect of gabapentinoids were included in this meta-analysis (Figure 7) [82,83]. Both studies explored the main outcomes at 24 and 48 h. No studies exploring the preventive effect of gabapentinoids on post-craniotomy pain at 6 and 12 h were identified. Shimony et al. investigated the analgesic effect of 150 mg of pregabalin [83], while Zeng et al. explored the analgesic effect of 600 mg of gabapentin administered the night before surgery and two hours before anesthesia induction [82]. The drugs were orally administered in both studies. The pooled estimates (MD) were −1.03 (CIs: −1.32; −0.74; *I*^2^ = 0%; *p* < 0.0001) at 24 h and −0.51 (CIs: −2.54; 1.52; *I*^2^ = 25%; *p* = 0.19) at 48 h.

- Subgroup Analysis

The patient population was constituted by both craniotomy location categories (supra- and infra-tentorial). However, subgroup analyses were not performed, as the outcome measures were not independently evaluated. Additionally, a subgroup analysis of different agents could not be conducted, as only two studies from the gabapentinoids class were identified.

#### 3.3.6. Agonist of Adrenal Receptors

**Figure 8:**
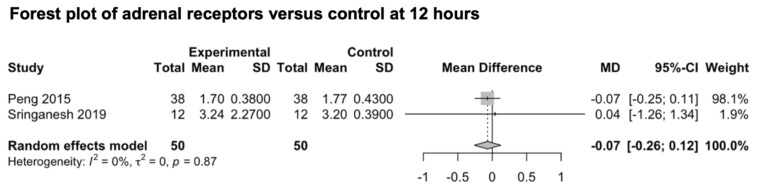
Forest plot of agonists of adrenal receptors versus control at 12 h [84,85].

We included two studies investigating the preventive effect of adrenal receptor agonists on post-craniotomy pain (Figure 8) [84,85]. The main outcome was reported for both studies at 24 h. Only one study reported the pain outcome at 6 and 12 h [84], and only one study reported the pain outcomes at 48 h [85]. The studies explored the analgesic effect of intravenously administered dexmedetomidine (0.5 μg/kg/h) [84,85]. The pooled estimate (MD) was −0.07 (CIs: −0.26; 0.12; *I*^2^ = 0%; *p* = 0.14).

- Subgroup Analysis

No subgroup analyses were conducted since all the included studies investigated the role of adrenal receptor agonists in a patient population that underwent supra-tentorial craniotomies.

#### 3.3.7. Combined Use of Agonists of Adrenal Receptors and Local Anesthetics in Preventive Local Treatments (Scalp Block and Scalp Incision Infiltration)

We included two studies investigating the combined analgesic value of adrenal receptors’ agonists and local anesthetics against local anesthetics alone for scalp block and scalp incision infiltration [86,87]. Senapathi et al. investigated the role of clonidine (2 μg/kg) together with levobupivacaine (0.25%) [86], while Vallapu et al. analyzed the role of dexmedetomidine (1 μg/kg) and bupivacaine (0.25%) [87]. The pooled estimate (MD) was not calculated since the study headed by Senapathi et al. was judged to be at a high risk of bias.

#### 3.3.8. Combined Use of Steroids and Local Anesthetics in Scalp Infiltration

**Figure 9:**
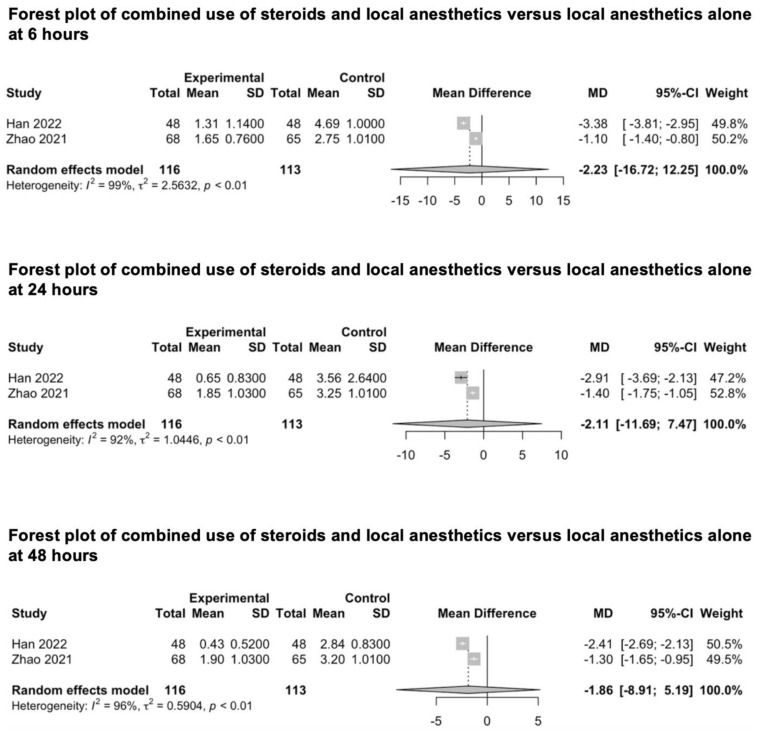
Forest plot of combined use of steroids and local anesthetics versus local anesthetics alone at 6, 24 and 48 h [88,89].

We included two studies investigating the combined analgesic value of steroids and local anesthetics against local anesthetics alone for scalp infiltration (Figure 9) [88,89]. Han et al. investigated the combined effects of diprospan (0.5 mL) and ropivacaine (0.5%) [88], while Zhao et al. investigated the combined effect of dexamethasone (10 mg) and ropivacaine (150 mg) [89]. The pooled estimates (MD) were −2.23 (CIs: −16.72; 12.25; *I*^2^ = 99%; *p* = 0.3) at 6 h, −2.11 (CIs: −11.69; 7.47; *I*^2^ = 92%; *p* = 0.21) at 24 h and −1.86 (CIs: −8.91; 5.19; *I*^2^ = 96%; *p* = 0.18) at 48 h.

- Subgroup Analysis

No subgroup analyses were conducted since all the included studies investigated the adjunctive role of steroids for scalp infiltration in a patient population of supra-tentorial craniotomies. Moreover, we were unable to conduct a subgroup analysis of the different agents since only two studies were identified.

#### 3.3.9. Intravenous Infusion of Lidocaine

We found only one study investigating the analgesic effect of intravenously administered lidocaine for the prevention of post-craniotomy pain [90]. The authors analyzed the effects of lidocaine, which was administered as a bolus of 1mg/kg, followed by the continuous infusion of 2 mg/kg/h. No pooled effects were calculated since no other studies were identified that investigated the analgesic effect of intravenous infusions of lidocaine or other different sodium channel blockers.

### 3.4. Sensitive Analyses

No sensitive analyses were conducted in this study since the pooled estimates calculated in the meta-analysis did not include studies that were judged to have a high risk of bias.

## 4. Discussion

The inadequate control of pain after craniotomy is a critical issue in the perioperative management of neurosurgical patients as it relates to worse surgical outcomes [91,92]. Agitation and the consequent activation of the sympathetic nervous system can cause complications during the primary post-operative period, leading to secondary neurological injury [6,93]. In the meantime, analgesic drugs can have significant side effects that can hinder patient recovery after neurosurgical intracranial procedures [6,94]. Therefore, the management of pain after craniotomies requires a balanced approach that is achievable through a tailored analgesic plan for patients with intracranial pathologies [50,95,96]. Although the importance of post-craniotomy pain management is well recognized, we found only one previous comprehensive meta-analysis investigating the pharmacological prevention treatments for acute pain in patients undergoing craniotomies [21]. The authors included the pooled estimates of NSAIDs, gabapentinoids, acetaminophen, scalp block and scalp infiltration, concluding with high-quality evidence that NSAIDs reduce pain for up to 24 h post-operatively. Meanwhile, they failed to find a beneficial effect with equal certainty of the evidence for the other investigated treatments. This could have been determined by the limited number of published RCTs up to that time. To this extent, the growing number of studies over the last three years (30% of our initially identified records) prompted the need for updating the existing knowledge on post-craniotomy pain management.

This meta-analysis included 29 RCTs, examining the preventive analgesic effect of NSAIDs, acetaminophen, scalp block, scalp infiltration, gabapentinoids, intravenously administered lidocaine and agonists of adrenal receptors through a direct comparison to control groups. Twelve studies (41%) were published between 2019 and 2022.

### 4.1. NSAIDs Compared to Control

The effectiveness of NSAIDs in reducing post-craniotomy pain for up to 24 h post-operatively is confirmed by the present investigation. The levels of evidence certainty (EC) were graded as moderate for the pooled estimates at 6 and 12 h and as high at 24 h. The small sample size and the indirectness caused by the employment of different anti-inflammatory agents downgraded the level of EC [62,63,64]. Conversely, it was increased following an identified dose–response gradient.

### 4.2. Acetaminophen Compared to Control

This meta-analysis failed to confirm the efficacy of acetaminophen in reducing post-craniotomy pain at 6 and 12 h after surgery with a moderate-to-low degree of EC. This result took into account a relatively low number of included patients and a moderate-to-high level of heterogeneity. Differently, there was *high* EC that acetaminophen has a sensible effect on reducing post-operative pain 24 h after surgery. This result was supported by a *low* degree of heterogeneity and an adequate sample size in the included studies.

### 4.3. Scalp Block Compared to Control

The efficacy of scalp block for post-craniotomy pain 6 h after surgery is suggested by the current investigation. This result is supported by a *moderate* degree of EC. The scalp block technique failed to be identified as effective for pain prevention at 12, 24, and 48 h after surgery. Nevertheless, this result has a *very low* degree of EC in consideration of the high level of inconsistency, indirectness and imprecision of the included studies and the high risk of reporting bias for the studies included in the pooled analysis at 24 h.

Subgroup analyses revealed a significant effect of ropivacaine in reducing post-craniotomy pain at 6 h after surgery with a *high* degree of EC. The same result failed to be confirmed at 24 h despite it having a *very low* EC level.

### 4.4. Scalp Infiltration Compared to Control

The present meta-analysis could not support the impact of scalp infiltration on reducing pain at 6, 12 and 24 h after craniotomy. Nevertheless, these results showed a *low* degree of EC since they were burdened by a serious level of imprecision related to the small sample size.

### 4.5. Gabapentinoids Compared to Control

The pooled estimates revealed gabapentinoids to be effective for post-craniotomy pain 24 h after surgery. Conversely, their efficacy failed to be confirmed at 48 h. However, these results must be considered with care because of a *low* degree of EC secondary to a remarkable level of indirectness due to the employment of different drugs (gabapentin and pregabalin) and some degrees of imprecision caused by a small sample size.

### 4.6. Other Interventions

The current meta-analysis failed to identify any positive effects of agonists of adrenal receptors on improving post-craniotomy pain compared to that of the control group. Likewise, no differences in post-craniotomy pain were demonstrated when the combination of steroids and local anesthetics were compared to local anesthetics alone. The level of EC was revealed to be *low* to *very low* due to significant unexplained heterogeneity, a limited cohort of patients and a high level of indirectness caused by different drugs. Finally, no pooled estimates could be calculated to measure the analgesic value of the intravenously administered lidocaine compared to that of the control group, and the one of adrenal receptors’ agonists combined with local anesthetics compared to local anesthetics alone for scalp block and scalp incision infiltration.

A “Summary of Findings” Table generated by GRADEpro GDT software is available as Table S3 (Supplementary Materials).

## 5. Conclusions

The present meta-analysis provides high-certainty evidence that NSAIDs and acetaminophen may have a more moderate effect on reducing post-craniotomy pain 24 h after surgery compared to that of the control groups. Further, there is high-certainty evidence suggesting ropivacaine scalp block may have a larger effect on reducing post-craniotomy pain 6 h after surgery compared to that of the control groups. Moderate-certainty evidence indicates NSAIDs may have a more noticeable effect on reducing post-craniotomy pain 12 h after surgery compared to that of the control group. No moderate-to-high-certainty evidence indicates effective treatments for post-craniotomy pain prevention 48 h after surgery.

## Supplementary Materials

The following supporting information can be downloaded at: https://www.mdpi.com/article/10.3390/medicina59050831/s1, Figure S1: Forest plots of scalp block subgroup analysis; Figure S2: Funnel plots for scalp block reporting bias analysis; Table S1: type of studies and population features. APAP: acetaminophen; BVC: bupivacaine; DEX: dexmedetomidine; LoS: length of stay; NR: not reported; Table S2: risk of bias for each included study according to the RoB2 revised tool for assessing the risk of bias in randomized trials. Table S3: Summary of Findings.
