# Supplementary material for: Prevention of Post-Operative Pain after Elective Brain Surgery: A Meta-Analysis of Randomized Controlled Trials
Source: Medicina (Kaunas). 2023 Apr 24;59(5):831. doi: 10.3390/medicina59050831 (PMC10220698; doi:10.3390/medicina59050831)
Supplement: Supplementary file 1 [file medicina-59-00831-s001.zip › Table S1.pdf]

Author	Number of participants	Pathology	Supra-tentorial	Infra-tentorial	ASA score	Drug class	Drug name	Administration route	Dose	Control group	Pain grading scale	Type of anesthesia	Evaluation of sedation	Follow-up
Greenberg 2018	131	Supra and infratentorial pathologies (unspecified)	70.8%	29.2%	III-IV	APAP	APAP	EV	1000 mg	Sham	VAS	General	Arterial blood pressure and heart rate	NR
Artime 2018	96	Supratentorial tumors	96	0	III (64-70%)	APAP	APAP	EV	1000 mg	Sham	VAS	General	Electrocardiogram and invasive blood pressure monitoring	24 hrs
Burbridge 2019	20	MoyaMoya disease	20	0	NR	APAP	APAP	EV	1000 mg	Sham	VAS	General	Electroencephalographic and evoked potential techniques	NR
Sivakumar 2018	204	Tumors, aneurysms, vascular malformations, and epilepsy	204	0	NR	APAP	APAP	EV	1000 mg	Sham	VAS	General	NR	48 hrs
Peng 2015	76	Brain tumors and intracranial vascular lesions	80	0	I-II	Alfa2ini	DEX	EV	0.5 mg/kg/h	Sham	NRS	General	Pulse oximetry, electro-cardiogram, arterial blood pressure, central venous pressure, and end-tidal carbon dioxide.	24 hrs
Sringanesh 2019	24	NR	24	0	NR	Alfa2ini	DEX	EV	0.5 µg/kg/h	Sham	NRS	General	Spectral entropy monitor	48 hrs
Mahajan 2019	27	Tumors	45	0	I-II	Local Anesthetic	Lidocaine	EV	Bolus of 1.5 mg/kg lignocaine over 15 min, followed by infusion of 2 mg/kg/h (6 ml/hr).	Sham	VAS	General	Electrocardiography, pulse oximetry and automated noninvasive blood pressure + BIS.	24 hrs
Jones 2008	80	NR	NR	NR	NR	NSAID	Parecoxib	EV	40 mg	Saline	VAS	General	NR	24 hrs
Zeng 2019	102	Tumors	87	15	I-II	Gabapentinoid	Gabapentin	OS	300 mg x 4	Sham (VitaminB)	VAS	General	Electrocardiograph, pulse oxygen saturation, continuous arterial pressure, end-tidal carbon dioxide partial	48 hrs

													pressure (ECO2), and bispectral index (BIS).	
Yadav 2014	249	NR	390	NR	I-II	NSAID	Diclofenac	OS	50 mg	Sham (Vitamin B)	VAS	General	NR	48 hrs
Molnar 2015	200	Tumors	165	35	NR	NSAID	Diclofenac	OS	100 mg	Nothing	VAS	General	NR	24 hrs
Shimony 2016	100	Tumors	87	13	I-III	Gabapentinoid	Pregabalin	OS	150 mg	Sham	NRS	General and awake	NR	72 hrs
Akcil 2017	30	Infratentorial tumors	0	47	I-III	Local Anesthetic	BVC	Scalp Block	6 ml 0.5%	Sham	VAS	General	NR	24 hrs
Rigamonti 2020	85	Tumor, aneurisms, AVMs and Cavernomas	89	0	NR	Local Anesthetic	BVC	Scalp Block	20 mL of 0.5% BVC with 1:200,000 epinephrine	Sham	VAS	General	Arterial waveform monitors and temperature probes	60 days
Can 2017	60	Aneurisms and other vascular diseases	NR	NR	I-II	Local Anesthetic	BVC and LevoBVC	Scalp Block	20 mL of 0.5% BVC (Group B), 20 mL of 0.5% levoBVC (Group L, n ¼ 30)	Sham	VAS	General	Electrocardiography and non-invasive blood pressure pressure measurement, and pulse oximetry	24 hrs
Vallapu 2018	100	NR	129	21	I-II	Local Anesthetic	BVC+DEX	Scalp Block	BVC (0.25%) and DEX (1 μg/kg)	BVC	NRS	General	Heart rate, systolic and diastolic blood pressure, respiration rate, and oxygen saturation (SpO2)	3 months
Carella 2021	60	Supratentorial intracranial surgery	60	0	I-III	Local Anesthetic	LevoBVC	Scalp Block	30 mL of 0.33%	Sham	NRS	General	Electrocardiogram, pulse oximetry, noninvasive blood pressure with adapted cuff size, end-tidal CO2, and state entropy of the electroencephalogram	48 hrs
Hwang 2015	46	Unruptured aneurisms	52	0	I-II	Local Anesthetic	LevoBVC	Scalp Block	7 mL of 0.75% levoBVC and 1:200,000 epinephrine.	Sham	NRS	General	Electrocardiography, pulse oximetry, and noninvasive blood pressure	72 hrs
Senapathi 2019	46	Tumors	NR	NR	I-III	Local Anesthetic	LevoBVC+Clonidine	Scalp Block	LevoBVC 0.25% and clonidine 2 μg/kg	LevoBVC	NRS	General	NR	24 hrs
Gazoni 2008	30	Supratentorial tumors	30	0	NR	Local Anesthetic	Ropivacaine	Scalp Block	30 ml of 0.5%	Nothing	VAS	General	Blood pressure and heart rate	48 hrs

Yang 2020	44	NR	44	0	I-II	Local Anesthetic	Ropivacaine	Scalp Block	10 ml of 0.2% to 0.5% ropivacaine	Sham	VAS	General	Electrocardiography, saturation of pulse oximetry, end-tidal carbon dioxide pressure, and invasive blood pressure measurement	24 hrs
Yang 2019	33	Aneurisms	57	0	I-II	Local Anesthetic	Ropivacaine	Scalp Block	15 mL of 0.75%	Nothing	VAS	General	Electrocardiography, pulse oximetry, noninvasive blood pressure, end-tidal CO2 (ETCO2), nasopharyngeal temperature, and bispectral index (BIS).	72 hrs
Kulikov 2021	56	Supratentorial tumors	56	0	NR	Local Anesthetic	Ropivacaine	Scalp Inf Pre	5-7 ml - 7.5 mg/ml	Scalp Inf Post	VAS	General	Pulse oximetry, 5-lead electrocardiogram, and noninvasive blood pressure	24
Saringcarinkul 2008	49	NR	50	0	I-III	Local Anesthetic	BVC+Adrenaline	Scalp infiltration	20 ml of 0.5% BVC + adrenaline 1:400,000	Sham	VNS	General	NR	12 hrs
Biswas 2003	41	Supratentorial tumors	41	0	I - II	Local Anesthetic	BVC+Adrenaline	Scalp Infiltration	25 mL of 0.25%	Sham	VAS	General	Heart rate, systolic blood pressure, diastolic blood pressure, and mean arterial pressure	48 HRS
Zhao 2021	133	NR	133	0	I-II	Local Anesthetic	Desametasone+ Ropivacaine	Scalp Infiltration	10 mg dexamethasone + 150 mg ropivacaine	Ropivacaine	NRS	General	Blood pressure, heart rate, electrocardiography, pulse oximetry (SpO2) and bispectral index (BIS)	48 hrs
Han 2022	96	Supratentorial lesions	96	0	I-III	Local Anesthetic	Betamethasone +Ropivacaine	Scalp Infiltration	0.5-mL Diprospan (Diprospan betamethasone 1 mL, propionate 5 mg, and betamethasone sodium phosphate 2 mg) and 15 mL of 1% ropivacaine	Ropivacaine	NRS	General	Blood pressure, heart rate, peripheral pulse oximetry, and electrocardiography	48 hours
Zhou 2016	106	NR	NR	NR	I-II	Local Anesthetic	Ropivacaine	Scalp infiltration	10 ml of 0.5 %	Saline	VAS	General	Electrocardiogram, noninvasive	3 months

													blood pressure and pulse oximetry	
Song 2015	52	Tumors, vascular lesions, other	60	0	I-III	Local Anesthetic	Ropivacaine + Lidocaine	Scalp Infiltration	0.5 % ropivacaine and 1 % lidocaine (40 ml)	Sham	NRS	General	Blood pressure, heart rate, electrocardiograms tracings, respiratory rate, and SpO2	24 hrs

Table S1: type of studies and population features. APAP: acetaminophen; BVC: bupivacaine; DEX: dexmedetomidine; LoS: length of stay; NR: not reported.
