# Supplementary material for: Prevention of Post-Operative Pain after Elective Brain Surgery: A Meta-Analysis of Randomized Controlled Trials
Source: Medicina (Kaunas). 2023 Apr 24;59(5):831. doi: 10.3390/medicina59050831 (PMC10220698; doi:10.3390/medicina59050831)
Supplement: Supplementary file 1 [file medicina-59-00831-s001.zip › Table S2.pdf]

Revised Cochrane Risk of Bias Analysis	Artime et al., 2018	Akcil et al., 2017	Biswas et al., 2003	Burbridge et al., 2019	Can et al., 2017	Carella et al., 2021	Gazoni et al., 2008	Greenberg et al., 2018	Han et al., 2022	Hwang et al., 2015	Jones et al., 2008	Kuikov et al. 2021	Mahajan et al., 2019	Molnar et al., 2015	Peng et al., 2015	Rigamonti et al., 2020	Saringcarinkul et al., 2008	Senapathi et al., 2019	Shimony et al., 2016	Sivakumar et al., 2019	Song et al., 2015	Sringanesh et al., 2019	Vallapu et al., 2018	Yadav et al., 2014	Yang et al., 2019	Yang et al., 2020	Zeng et al., 2019	Zhao et al., 2021	Zhou et al., 2016
**Bias arising from the randomization process**	+	?	+	+	+	+	+	+	+	+	+	+	+	+	+	+	+	-	+	+	-	+	+	+	+	?	+	+	+
**Bias due to deviations from the intended interventions (effect of assignment to intervention)**	+	?	+	+	+	+	?	+	?	+	+	+	+	+	+	+	+	-	+	+	-	+	+	+	+	+	+	+	+
**Bias due to deviations from the intended interventions (effect of adhering to intervention)**	+	?	+	+	+	+	?	+	?	+	+	+	+	+	+	+	+	-	+	+	-	+	+	+	+	+	+	+	+
**Missing outcome data**	+	?	+	+	+	?	+	?	?	?	+	+	+	+	+	+	+	+	+	+	+	+	+	+	+	+	+	+	+
**Bias in measurement of the outcome**	+	+	+	+	+	?	+	+	+	+	?	+	?	+	+	+	+	?	+	+	?	+	+	+	+	+	+	+	+
**Bias in selection of the reported result**	+	+	+	+	+	+	+	+	+	+	+	+	+	+	+	+	+	?	+	+	?	+	+	+	+	+	+	+	+
**Overall risk of bias**	+	?	+	+	+	+	+	+	?	+	?	+	+	+	+	+	+	-	+	+	-	+	+	+	+	?	+	+	+

Table S2: risk of bias for each included study according to the RoB2 revised tool for assessing the risk of bias in randomized trials.
