# Supplementary material for: Prevention of Post-Operative Pain after Elective Brain Surgery: A Meta-Analysis of Randomized Controlled Trials
Source: Medicina (Kaunas). 2023 Apr 24;59(5):831. doi: 10.3390/medicina59050831 (PMC10220698; doi:10.3390/medicina59050831)
Supplement: Supplementary file 1 [file medicina-59-00831-s001.zip › Table S3.pdf]

Question: NSAIDs compared to Control for Post-Craniotomy Pain
Setting: RCTs

Certainty assessment							№ of patients		Effect		Certainty	Importance
№ of studies	Study design	Risk of bias	Inconsistency	Indirectness	Imprecision	Other considerations	NSAIDs	Control	Relative (95% CI)	Absolute (95% CI)		
**Post-Craniotomy Pain 6 hours (follow-up: mean 6 hours; Scale from: 0 to 10)**												
2	randomised trials	not serious	not serious	serious[b]	serious[a]	dose response gradient	166	163	-	MD **1.04 lower** (1.39 lower to 0.69 lower)	⨁⨁⨁◯ Moderate	
**Post-Craniotomy Pain at 12 hours (follow-up: mean 12 hours; Scale from: 0 to 10)**												
2	randomised trials	not serious	not serious	serious[b]	serious[a]	dose response gradient	166	163	-	MD **0.83 lower** (1.47 lower to 0.2 lower)	⨁⨁⨁◯ Moderate	
**Post-Craniotomy Pain at 24 hours (follow-up: mean 24 hours; Scale from: 0 to 10)**												
3	randomised trials	not serious	not serious	serious[b]	not serious	dose response gradient	266	263	-	MD **1.01 lower** (1.75 lower to 0.26 lower)	⨁⨁⨁⨁ High	

CI: confidence interval; **MD:** mean difference

Explanations

a. The imprecision was rated as serious because of a small sample size.
b. We rated the indirectness as serious because of the use of different drugs.

Question: Acetaminophen compared to Control for Post-Craniotomy Pain
Setting: RCTs

Certainty assessment							№ of patients		Effect		Certainty	Importance
№ of studies	Study design	Risk of bias	Inconsistency	Indirectness	Imprecision	Other considerations	Acetaminophen	Control	Relative (95% CI)	Absolute (95% CI)		
**Post-Craniotomy Pain at 6 hours (follow-up: mean 6 hours; Scale from: 0 to 10)**												
3	randomised trials	not serious	serious[a]	not serious	not serious	none	213	208	-	MD **0.59 lower** (2.06 lower to 0.89 higher)	⨁⨁⨁◯ Moderate	
**Post-Craniotomy Pain at 12 hours (follow-up: mean 12 hours; Scale from: 0 to 10)**												
2	randomised trials	not serious	serious[a]	not serious	serious[b]	none	147	143	-	MD **0.38 lower** (4.7 lower to 3.94 higher)	⨁⨁◯◯ Low	
**Post-Craniotomy Pain at 24 hours (follow-up: mean 24 hours; Scale from: 0 to 10)**												
4	randomised trials	not serious	not serious	not serious	not serious	none	223	218	-	MD **0.95 lower** (1.5 lower to 0.4 lower)	⨁⨁⨁⨁ High	

CI: confidence interval; **MD:** mean difference

Explanations

a. We rated the Inconsistency as serious because of unexplained heterogeneity.
b. We rated the imprecision as serious because of the small sample size.

Question: Scalp Block compared to Control for Post-Craniotomy Pain
Setting: RCTs

Certainty assessment							№ of patients		Effect		Certainty	Importance
№ of studies	Study design	Risk of bias	Inconsistency	Indirectness	Imprecision	Other considerations	Scalp Block	Control	Relative (95% CI)	Absolute (95% CI)		
**Post-Craniotomy Pain at 6 hours (Scale from: 0 to 10)**												
8	randomised trials	not serious	serious[a]	serious[b]	not serious	dose response gradient	188	190	-	MD **1.16 lower** (1.89 lower to 0.84 lower)	⊕⊕⊕◯ Moderate	
**Post-Craniotomy Pain at 12 hours (Scale from: 0 to 10)**												
4	randomised trials	not serious	serious[a]	serious[b]	serious[c]	none	95	99	-	MD **1.11 lower** (2.86 lower to 0.64 higher)	⊕◯◯◯ Very low	
**Post-Craniotomy Pain at 24 hours (Scale from: 0 to 10)**												
7	randomised trials	not serious	serious[a]	serious[b]	not serious	publication bias strongly suspected	174	174	-	MD **1.1 lower** (2.26 lower to 0.07 higher)	⊕◯◯◯ Very low	
**Post-Craniotomy Pain at 48 hours (Scale from: 0 to 10)**												
4	randomised trials	not serious	serious[a]	serious[b]	serious[c]	none	107	107	-	MD **1.48 lower** (3.85 lower to 0.9 higher)	⊕◯◯◯ Very low	

CI: confidence interval; **MD:** mean difference

Explanations

a. We rated the inconsistency as serious because of the unexplained heterogeneity.
b. We rated the indirectness as serious because of the use of different drugs.
c. We rated the imprecision as serious because of the small sample size.

Question: Bupivacaine - Scalp Block compared to Control for Post-Craniotomy Pain
Setting: RCTs

Certainty assessment							№ of patients		Effect		Certainty	Importance
№ of studies	Study design	Risk of bias	Inconsistency	Indirectness	Imprecision	Other considerations	Bupivacaine - Scalp Block	Control	Relative (95% CI)	Absolute (95% CI)		
**Post-Craniotomy Pain at 6 hours (Scale from: 0 to 10)**												
3	randomised trials	not serious	not serious	not serious	serious[b]	none	86	89	-	MD **0.23 lower** (1.07 lower to 0.61 higher)	⨁⨁⨁◯ Moderate	
**Post-Craniotomy Pain at 24 hours (Scale from: 0 to 10)**												
3	randomised trials	not serious	serious[a]	not serious	serious[b]	none	86	89	-	MD **0.13 lower** (2.03 lower to 1.78 higher)	⨁⨁◯◯ Low	

CI: confidence interval; **MD:** mean difference

Explanations

a. We rated the inconsistency as serious because of the unexplained heterogeneity.
b. We rated the imprecision as serious because of the small sample size.

Question: Levobupivacaine - Scalp Block compared to Control for Post-Craniotomy Pain
Setting: RCTs

Certainty assessment							№ of patients		Effect		Certainty	Importance
№ of studies	Study design	Risk of bias	Inconsistency	Indirectness	Imprecision	Other considerations	Levobupivacaine - Scalp Block	Control	Relative (95% CI)	Absolute (95% CI)		
**Post-Craniotomy Pain at 6 hours (Scale from: 0 to 10)**												
2	randomised trials	not serious	not serious	not serious	serious[a]	none	50	46	-	MD **2.41 lower** (5.38 lower to 0.57 higher)	⨁⨁⨁◯ Moderate	
**Post-Craniotomy Pain at 24 hours (Scale from: 0 to 10)**												
2	randomised trials	not serious	not serious	not serious	serious[a]	none	50	46	-	MD **2.39 lower** (4.79 lower to 0.02 higher)	⨁⨁⨁◯ Moderate	

CI: confidence interval; **MD:** mean difference

Explanations

a. We rated the imprecision as serious because of the small sample size.

Question: Ropivacaine - Scalp Block compared to Control for Post-Craniotomy Pain
Setting: RCTs

Certainty assessment							№ of patients		Effect		Certainty	Importance
№ of studies	Study design	Risk of bias	Inconsistency	Indirectness	Imprecision	Other considerations	Ropivacaine - Scalp Block	Control	Relative (95% CI)	Absolute (95% CI)		
**Post-Craniotomy Pain at 6 hours (Scale from: 0 to 10)**												
3	randomised trials	not serious	not serious	not serious	serious[b]	strong association	52	55	-	MD **1.31 lower** (1.68 lower to 0.95 lower)	⨁⨁⨁⨁ High	
**Post-Craniotomy Pain at 24 hours (Scale from: 0 to 10)**												
2	randomised trials	not serious	very serious[a]	not serious	serious[b]	none	38	39	-	MD **1.52 lower** (14.29 lower to 11.25 higher)	⨁◯◯◯ Very low	

CI: confidence interval; **MD:** mean difference

Explanations

a. We rated the inconsistency as serious because of the unexplained heterogeneity.
b. We rated the imprecision as serious because of the small sample size.

Question: Scalp Infiltration compared to Control for Post-Craniotomy Pain
Setting: RCTs

Certainty assessment							№ of patients		Effect		Certainty	Importance
№ of studies	Study design	Risk of bias	Inconsistency	Indirectness	Imprecision	Other considerations	Scalp Infiltration	Control	Relative (95% CI)	Absolute (95% CI)		
**Post-Craniotomy Pain at 6 hours (follow-up: mean 6 hours; Scale from: 0 to 10)**												
3	randomised trials	not serious	not serious	serious[a]	serious[b]	none	98	98	-	SMD **0.12 SD lower** (0.36 lower to 0.11 higher)	⨁⨁◯◯ Low	
**Post-Craniotomy Pain at 12 hours (follow-up: mean 12 hours; Scale from: 0 to 10)**												
2	randomised trials	not serious	not serious	serious[a]	serious[b]	none	45	45	-	SMD **0.14 SD lower** (2.08 lower to 1.8 higher)	⨁⨁◯◯ Low	
**Post-Craniotomy Pain at 24 hours (follow-up: mean 24 hours; Scale from: 0 to 10)**												
2	randomised trials	not serious	not serious	serious[a]	serious[b]	none	74	73	-	MD **0.02 higher** (0.09 lower to 0.12 higher)	⨁⨁◯◯ Low	

CI: confidence interval; **SMD:** standardised mean difference

Explanations

a. We graded the indirectness as serious because of the use of different drugs.
b. We graded the imprecision as serious because of the small sample size.

Question: Gabapentinoids compared to Control for Post-Craniotomy Pain
Setting: RCTs

Certainty assessment							№ of patients		Effect		Certainty	Importance
№ of studies	Study design	Risk of bias	Inconsistency	Indirectness	Imprecision	Other considerations	Gabapentinoids	Control	Relative (95% CI)	Absolute (95% CI)		
**Post-Craniotomy Pain at 24 hours (Scale from: 0 to 10)**												
2	randomised trials	not serious	not serious	serious[a]	serious[b]	none	102	100	-	MD **1.03 lower** (1.32 lower to 0.74 lower)	⨁⨁◯◯ Low	
**Post-Craniotomy Pain at 48 hours (Scale from: 0 to 10)**												
2	randomised trials	not serious	not serious	serious[a]	serious[b]	none	102	100	-	MD **0.51 lower** (2.54 lower to 1.52 higher)	⨁⨁◯◯ Low	

CI: confidence interval; **MD:** mean difference

Explanations

a. We graded the indirectness as serious because of the use of different drugs.
b. We graded the imprecision as serious because of the small sample size.

Question: Agonists of Adrenal Receptors compared to Control for Post-Craniotomy Pain
Setting: RCTs

Certainty assessment							№ of patients		Effect		Certainty	Importance
№ of studies	Study design	Risk of bias	Inconsistency	Indirectness	Imprecision	Other considerations	Agonists of Adrenal Receptors	Control	Relative (95% CI)	Absolute (95% CI)		
**Post-Craniotomy Pain at 12 hours (Scale from: 0 to 10)**												
2	randomised trials	not serious	not serious	not serious	serious[c]	none	50	50	-	MD **0.07 lower** (-0.26 lower to 0.12 higher)	⨁⨁⨁◯ Moderate	

CI: confidence interval; **MD:** mean difference

Explanations

a. We rated the inconsistency as serious because of the unexplained heterogeneity.
b. We rated the indirectness as serious because of the use of different drugs.
c. We rated the imprecision as serious because of the small sample size.

Question: Steroids and Local Anaesthetics compared to Local Anaesthetics Alone for Post-Craniotomy Pain at 6 hours
Setting:

Certainty assessment							№ of patients		Effect		Certainty	Importance
№ of studies	Study design	Risk of bias	Inconsistency	Indirectness	Imprecision	Other considerations	Steroids and Local Anaesthetics	Local Anaesthetics Alone	Relative (95% CI)	Absolute (95% CI)		
**Post-Craniotomy Pain at 6 hours (Scale from: 0 to 10)**												
2	randomised trials	not serious	very serious[a]	serious[b]	serious[c]	none	116	113	-	MD **2.23 lower** (16.72 lower to 12.25 higher)	⨁◯◯◯ Very low	
**Post-Craniotomy Pain at 24 hours (Scale from: 0 to 10)**												
2	randomised trials	not serious	very serious[a]	serious[b]	serious[c]	none	116	113	-	MD **2.11 lower** (11.69 lower to 7.47 higher)	⨁◯◯◯ Very low	
**Post-Craniotomy Pain at 48 hours (Scale from: 0 to 10)**												
2	randomised trials	not serious	very serious[a]	serious[b]	serious[c]	none	116	113	-	MD **1.86 lower** (8.91 lower to 5.19 higher)	⨁◯◯◯ Very low	

CI: confidence interval; **MD:** mean difference

Explanations

a. We rated the inconsistency as serious because of the unexplained heterogeneity.
b. We rated the indirectness as serious because of the use of different drugs.
c. We rated the imprecision as serious because of the small sample size.
