# Supplementary figures and images for: Prevention of Post-Operative Pain after Elective Brain Surgery: A Meta-Analysis of Randomized Controlled Trials
Source: Medicina (Kaunas). 2023 Apr 24;59(5):831. doi: 10.3390/medicina59050831 (PMC10220698; doi:10.3390/medicina59050831)

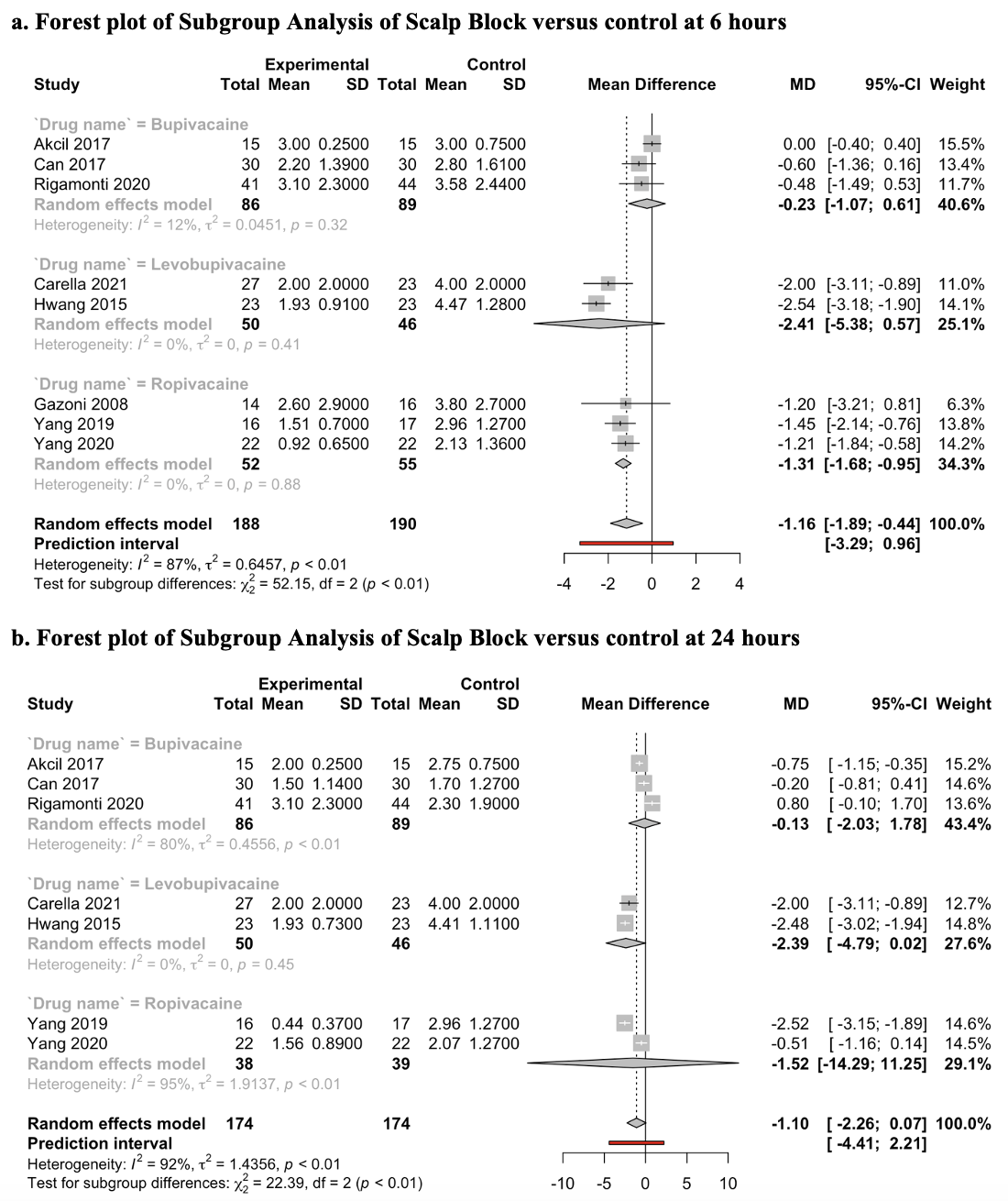

Supplement: Supplementary file 1 [file medicina-59-00831-s001.zip › Figure S1.tiff]

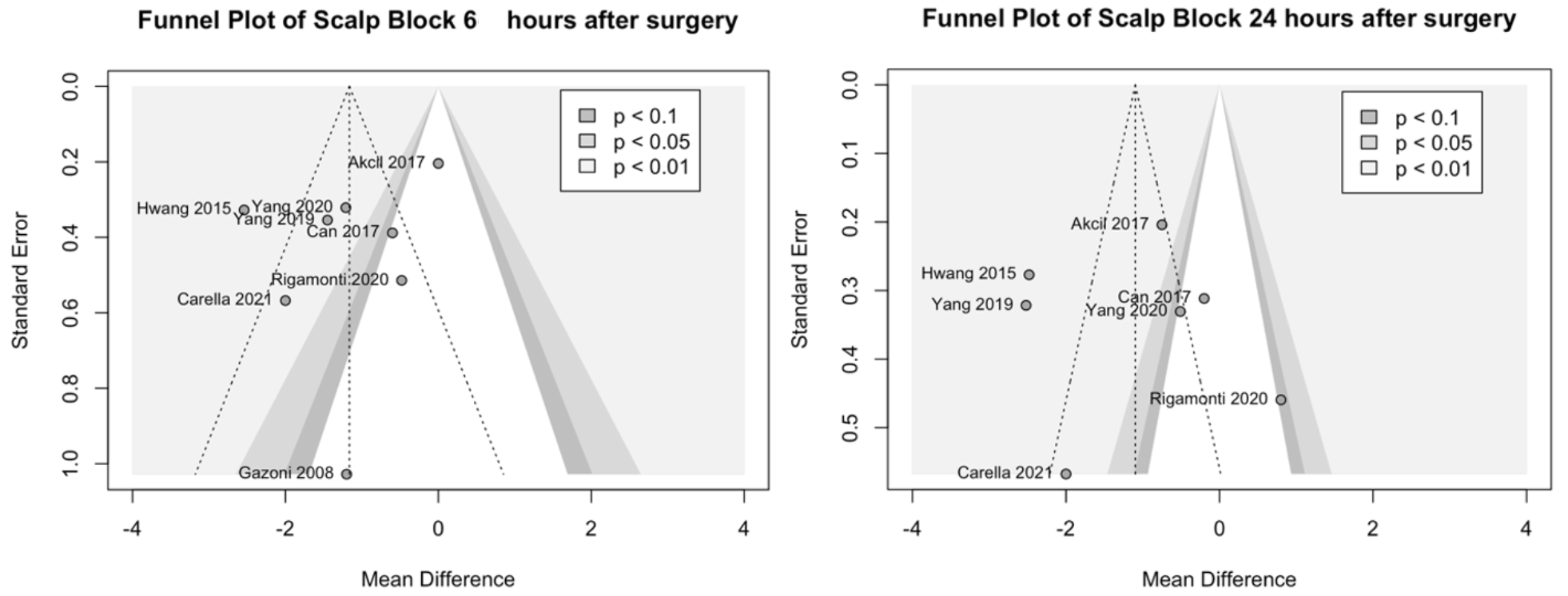

Supplement: Supplementary file 1 [file medicina-59-00831-s001.zip › Figure S2.tiff]
